# Supplementary material for: Co-circulation of all the four Dengue virus serotypes during 2018–2019: first report from Eastern Uttar Pradesh, India
Source: PeerJ. 2023 Jan 9;11:e14504. doi: 10.7717/peerj.14504 (PMC9835713; doi:10.7717/peerj.14504)
Supplement: Supplemental Information 2 — The phylogenetic tree constructed for DENV1-4 serotypes with Maximum Likelihood method and Tamura-Nei model using 1000 bootstrap value. The sequences from this study were highlighted with the red circle. Evolutionary analyses were conducted in MEGA X. [file peerj-11-14504-s002.docx]

**Supplementary Figure S1**: **The Cladogram for DENV1-serotypes based on CprM sequences.** The phylogenetic tree constructed for DENV-1 serotypes with Maximum Likelihood method and Tamura-Nei model using 1000 bootstrap value. The sequences from this study were highlighted with the red circle. Evolutionary analyses were conducted in MEGA X.

**Supplementary Figure S2:** : **The Cladogram for DENV2-serotypes based on CprM sequences.** The phylogenetic tree constructed for DENV-2 serotypes with Maximum Likelihood method and Tamura-Nei model using 1000 bootstrap value. The sequences from this study were highlighted with the red circle. Evolutionary analyses were conducted in MEGA X.

**Supplementary Figure S3:** : **The Cladogram for DENV3-serotypes based on CprM sequences.** The phylogenetic tree constructed for DENV-3 serotypes with Maximum Likelihood method and Tamura-Nei model using 1000 bootstrap value. The sequences from this study were highlighted with the red circle. Evolutionary analyses were conducted in MEGA X.

**Supplementary Figure S4:** : **The Cladogram for DENV4-serotypes based on CprM sequences.** The phylogenetic tree constructed for DENV-4 serotypes with Maximum Likelihood method and Tamura-Nei model using 1000 bootstrap value. The sequences from this study were highlighted with the red circle. Evolutionary analyses were conducted in MEGA X.
