# Supplementary material for: Co-circulation of all the four Dengue virus serotypes during 2018–2019: first report from Eastern Uttar Pradesh, India
Source: PeerJ. 2023 Jan 9;11:e14504. doi: 10.7717/peerj.14504 (PMC9835713; doi:10.7717/peerj.14504)
Supplement: Supplemental Information 4 [file peerj-11-14504-s004.docx]

**Supplementary Table 1**: The List of isolates sequences with their respective DENV serotype identified and GenBank accession number and Patient clinical detail.

| S. No. | GenBank accession number | Isolate number | DENV serotype | DENGUE IgM | DENGUE IgG | AGE | GENDER |
| --- | --- | --- | --- | --- | --- | --- | --- |
| 1 | MZ490477 | 1821035 | DENV-1 | Negative | Negative | 18 | M |
| 2 | MZ490478 | 1821237 | DENV-1 | Negative | Negative | 50 | F |
| 3 | MZ490479 | 1821047 | DENV-1 | Negative | Positive | 29 | M |
| 4 | MZ490480 | 1821067 | DENV-1 | Negative | Negative | 32 | M |
| 5 | MZ490481 | 1821080 | DENV-1 | Negative | Negative | 62 | M |
| 6 | MZ490482 | 1821082 | DENV-1 | Negative | Negative | 32 | M |
| 7 | MZ490483 | 1921118 | DENV-1 | Positive | Negative | 30 | M |
| 8 | MZ490484 | 1921253 | DENV-1 | Negative | Negative | 19 | M |
| 9 | MZ490485 | 1821071 | DENV-1 | Negative | Positive | 43 | M |
| 10 | MZ490486 | 1821017 | DENV-1 | Negative | Negative | 33 | F |
| 11 | MZ490487 | 1921207 | DENV-2 | Negative | Negative | 32 | M |
| 12 | MZ490488 | 1821036 | DENV-2 | Negative | Negative | 17 | M |
| 13 | MZ490489 | 1821090 | DENV-2 | Negative | Negative | 45 | F |
| 14 | MZ490490 | 1821103 | DENV-2 | Negative | Negative | 22 | M |
| 15 | MZ490491 | 1821013 | DENV-2 | Positive | Positive | 30 | M |
| 16 | MZ490492 | 1821014 | DENV-2 | Negative | Negative | 32 | F |
| 17 | MZ490493 | 1821025 | DENV-2 | Positive | Positive | 25 | M |
| 18 | MZ490494 | 1821032 | DENV-2 | Positive | Positive | 24 | M |
| 19 | MZ490495 | 1821038 | DENV-2 | Negative | Negative | 50 | M |
| 20 | MZ490496 | 1821046 | DENV-2 | Negative | Negative | 19 | M |
| 21 | MZ490497 | 1821047 | DENV-2 | Negative | Positive | 55 | M |
| 22 | MZ490498 | 1821049 | DENV-2 | Positive | Negative | 42 | M |
| 23 | MZ490499 | 1821056 | DENV-2 | Negative | Negative | 57 | F |
| 24 | MZ490500 | 1821068 | DENV-2 | Positive | Positive | 45 | M |
| 25 | MZ490501 | 1921005 | DENV-2 | Negative | Negative | 19 | M |
| 26 | MZ490502 | 1921006 | DENV-2 | Negative | Negative | 28 | M |
| 27 | MZ490503 | 1921016 | DENV-2 | Negative | Negative | 20 | M |
| 28 | MZ490504 | 1921019 | DENV-2 | Negative | Negative | 15 | M |
| 29 | MZ490505 | 1921022 | DENV-2 | Negative | Negative | 21 | M |
| 30 | MZ490506 | 1921044 | DENV-2 | Positive | Negative | 27 | M |
| 31 | MZ490507 | 1821074 | DENV-2 | Positive | Positive | 23 | M |
| 32 | MZ490508 | 1821079 | DENV-2 | Negative | Negative | 18 | F |
| 33 | MZ490509 | 1821081 | DENV-2 | Negative | Positive | 21 | M |
| 34 | MZ490510 | 1921120 | DENV-2 | Positive | Negative | 18 | F |
| 35 | MZ490511 | 1921257 | DENV-2 | Negative | Negative | 20 | M |
| 36 | MZ490512 | 1921175 | DENV-2 | Negative | Negative | 04 | F |
| 37 | MZ490513 | 1821101 | DENV-2 | Negative | Negative | 34 | M |
| 38 | MZ490514 | 1921008 | DENV-3 | Positive | Negative | 37 | M |
| 39 | MZ490515 | 1821077 | DENV-3 | Negative | Negative | 20 | M |
| 40 | MZ490516 | 1821070 | DENV-3 | Negative | Negative | 24 | F |
| 41 | MZ490517 | 1921239 | DENV-3 | Positive | Negative | 42 | M |
| 42 | MZ490518 | 1821045 | DENV-3 | Negative | Negative | 25 | F |
| 43 | MZ490519 | 1821021 | DENV-3 | Negative | Negative | 21 | M |
| 44 | MZ490520 | 1821010 | DENV-3 | Positive | Negative | 42 | M |
| 45 | MZ490521 | 1821050 | DENV-3 | Positive | Positive | 28 | M |
| 46 | MZ490522 | 1921039 | DENV-3 | Positive | Negative | 17 | M |
| 47 | MZ490523 | 1921042 | DENV-3 | Negative | Negative | 50 | M |
| 48 | MZ490524 | 1821072 | DENV-3 | Negative | Negative | 32 | M |
| 49 | MZ490525 | 1821085 | DENV-3 | Negative | Negative | 27 | F |
| 50 | MZ490526 | 1921126 | DENV-3 | Negative | Negative | 45 | F |
| 51 | MZ490527 | 1921159 | DENV-3 | Negative | Negative | 19 | M |
| 52 | MZ490528 | 1921223 | DENV-3 | Negative | Negative | 21 | M |
| 53 | MZ490529 | 1821080 | DENV-3 | Negative | Negative | 35 | F |
| 54 | MZ490530 | 1821029 | DENV-3 | Positive | Positive | 22 | M |
| 55 | MZ490531 | 1821057 | DENV-3 | Negative | Negative | 70 | M |
| 56 | MZ490532 | 1821051 | DENV-3 | Negative | Negative | 45 | M |
| 57 | MZ490533 | 1921087 | DENV-3 | Negative | Negative | 25 | M |
| 58 | MZ490534 | 1921105 | DENV-4 | Negative | Negative | 20 | M |
| 59 | MZ490535 | 1821086 | DENV-4 | Negative | Negative | 19 | M |
| 60 | MZ490536 | 1921011 | DENV-4 | Negative | Negative | 22 | M |
| 61 | MZ490537 | 1821089 | DENV-4 | Negative | Negative | 41 | M |
